# Erratum

**DOI:** 10.1002/jev2.12313

**Published:** 2023-03-14

Correction to “Glioblastoma‐derived extracellular vesicle subpopulations following 5‐aminolevulinic acid treatment bear diagnostic implications”

Hsia, T., Yekula, A., Batool, S. M., Rosenfeld, Y. B., You, D. G., Weissleder, R., Lee, H., Carter, B.S., & Balaj, L. (2022). Glioblastoma‐derived extracellular vesicle subpopulations following 5‐aminolevulinic acid treatment bear diagnostic implications. *Journal of Extracellular Vesicles*, 11(11), e12278. https://doi.org/10.1002/jev2.12278

The first published version of this paper did not list the below information for co‐authors.

Hsia and Yekula are co‐first authors.

We apologize for this error.

